# Population genetic data of the 21 autosomal STRs included in GlobalFiler kit of a population sample from the Kingdom of Bahrain

**DOI:** 10.1371/journal.pone.0220620

**Published:** 2019-08-15

**Authors:** Noora R. Al-Snan, Safia Messaoudi, Saranya R. Babu, Moiz Bakhiet

**Affiliations:** 1 Department of Molecular Medicine, College of Medical and Medicine Sciences, Arabian Gulf University, Kingdom of Bahrain; 2 Forensic Science Laboratory, Directorate of Forensic Science, General Directorate of Criminal Investigation and Forensic Science, Ministry of Interior, Kingdom of Bahrain; 3 Forensic Biology Department, College of Forensic Sciences, Naif Arab University for Security Sciences, Riyadh, Saudi Arabia; King Saud University, SAUDI ARABIA

## Abstract

Bahrain’s population consists mainly of Arabs, Baharna and Persians leading Bahrain to become ethnically diverse. The exploration of the ethnic origin and genetic structure within the Bahraini population is fundamental mainly in the field of population genetics and forensic science. The purpose of the study was to investigate and conduct genetic studies in the population of Bahrain to assist in the interpretation of DNA-based forensic evidence and in the construction of appropriate databases. 24 short-tandem repeats in the GlobalFiler PCR Amplification kit including 21 autosomal STR loci and three gender determination loci were amplified to characterize different genetic and forensic population parameters in a cohort of 543 Bahraini unrelated healthy men. Samples were collected during the year 2017. The genotyping of the 21 autosomal STRs showed all of the loci were in Hardy-Weinberg Equilibrium (HWE) after applying Bonferroni’s correction. We also found out no significant deviations from LD between pairwise STR loci in Bahraini population except when plotting for D3S1358-CSF1PO, CSF1PO-SE33, D19S433-D12S391, FGA-D2S1338, FGA-SE33, FGA-D7S820 and D7S820-SE33. The SE33 locus was the most polymorphic for the studied population and THO1 locus was the less polymorphic. The Allele 8 in TPOX scored the highest allele frequency of 0.496. The SE33 locus showed the highest power of discrimination (PD) in Bahraini population, whereas TPOX showed the lowest PD value. The 21 autosomal STRs showed a value of combined match probability (CMP) equal to 4.5633^E-27^, and a combined power of discrimination (CPD) of 99.99999999%. Off-ladders and tri-allelic variants were observed in various samples at D12S391, SE33 and D22S1045 loci. Additionally, pairwise genetic distances based on FST were calculated between Bahraini population and other populations extracted from the literature. Genetic distances were represented in a non-metric MDS plot and clustering of populations according to their geographic locations was detected. Phylogenetic tree was constructed to investigate the genetic relatedness between Bahraini population and the neighboring populations. Our study indicated that the twenty-one autosomal STRs are highly polymorphic in the Bahraini population and can be used as a powerful tool in forensics and population genetic analyses including paternity testing and familial DNA searching.

## Introduction

Kingdom of Bahrain is a small archipelago consisting of 33 islands, only the five largest are inhabited. These islands are Bahrain, Muharraq, Umm and Nasan and Sitra. Bahrain is positioned in the Arabian Gulf. To the southeast of Bahrain is the State of Qatar, and to its west lies the Kingdom of Saudi Arabia, with which it is connected by a 25-kilometer causeway. To the north and east of Bahrain lies the Islamic Republic of Iran **[1].**

Bahrain is one of the most densely populated countries in the world, with a total landmass of 760 square kilometers. Mid-2014, estimates of Bahrain’s population stood at 1,314,562 persons. Of these, 568,399 are Bahraini citizens (46%) and 666,172 are expatriates (54%) **[2].**

Standing between the most substantial focal points of the ancient world–the Far East, the Indus Valley, Fertile Crescent, the Red Sea and the Coast of East Africa **[3]**, trade goods from the Persian Gulf made its way into Europe through Antioch **[4].** This made Bahrain an important port city, a metropolitan hub where different cultures met **[5].**

Because of the geographic location of Bahrain, the diversity of the population had been affected. This could be explained by the migration flows from several areas regionally, and eventually internationally **[6].** Iranians and migrants of Iranian heritage constituted the largest groups of migrants who were Muslim and ethnically not Arab **[7]**. Indian and Iranian migration boomed in the early and mid-20th century, as the Bahrain Petroleum Company sought a workforce for the oil that was discovered in the island **[8].**

Population is mainly divided into four main ethnic groups: Arabs, Baharna and Persians (Huwala and Ajam) **[4,9,10].** This geographical and social organization might be expected to have an effect on patterns of a genetic diversity **[11].**

Genetic studies on Bahrain to date are very limited and knowledge of any such structure is important in the interpretation of the significance of DNA-based forensic evidence and in the construction of appropriate databases. This present study is the first to characterize genetically the Bahraini population, using Globalfiler amplification kit. Twenty-four autosomal short-tandem repeats (STRs) in GlobalFiler PCR Amplification kit (Thermo Fisher Scientific, Inc., Waltham, MA, USA) were studied to characterize different forensic and genetic population parameters in 545 Bahraini males. The 6-dye GlobalFiler PCR Amplification kit (Thermo Fisher Scientific, Inc., Waltham, MA, USA) was designed to incorporate 21 commonly used autosomal STR loci (D8S1179, D21S11, D7S820, CSF1PO, D3S1358, TH01, D13S317, D16S539, D2S1338, D19S433, VWA, TPOX, D18S51, D5S818, FGA, D12S391, D1S1656, D2S441, D10S1248, D22S1045 and SE33) and three gender determination loci (Amelogenin, Yindel and DYS391) which have been proven to provide reliable DNA typing results and enhance the power of discrimination (PD).

## Materials and methods

### Sample collection

Five hundred and forty-three (543) blood samples were collected on Nucleic-Cards (Copan, Italy) from non-relatives’ Bahraini males. The research study was announced publicly through different social media channels such as Twitter and Instagram. Participants who wished to participate contacted the corresponding author for establishing meetings and arrived to the General Directorate of Criminal investigation and Forensic Science–Kingdom of Bahrain to submit their blood samples for the research. Age of the participants ranged from 20 to 55 years old.

In each case, males with ancestry (to the level of paternal grandfather) from four different geographical subdivisions of the country (Capital Governorate, Muharraq Governorate, Northern Governorate and Southern Governorate) were sampled. Ethical review for analysis was provided and approved by the Research and Research Ethics Committee (RREC) (E007-PI-10/17) in the Arabian Gulf University. All participants provided informed consent for contribution their blood samples.

### DNA amplification and fragment detection

DNAs were punched and amplified from Nucleic-Cards (Copan, Italy) blood-spot samples using a fully automated workstation, starting from 1.2-mm diameter punches produced using the easyPunch STARlet system (Hamilton, Switzerland).

The samples were directly amplified using GlobalFiler (Thermo Fisher Scientific, Inc., Waltham, MA, USA) according to manufacturer’s recommendation. 15μl of low TE Buffer (pH 8.0) was added to the MicroAmp Optical 96-Well Reaction Plate (Thermo Fisher Scientific, Inc., Waltham, MA, USA) prior to the addition of 10μl of GlobalFiler master mix. A total of 24 loci were amplified, including 21 autosomal STR loci and three gender determination loci.

The PCR products (1μl) were separated by capillary electrophoresis in an ABI 3500xl Genetic Analyzer (Thermo Fisher Scientific Company, Carlsbad, USA) with reference to the LIZ600 size standard v2 (Thermo Fisher Scientific, Inc., Waltham, MA, USA) in total of 9 μl of LIZ600 standard and Hi-Di formamide (Thermo Fisher Scientific, Inc., Waltham, MA, USA) master mix. GeneMapper ID-X Software v1.4 (Thermo Fisher Scientific, Inc., Waltham, MA, USA) was used for genotype assignment. DNA typing and assignment of nomenclature were based on the ISFG recommendations.

### Statistical analysis

Allele frequencies, Minor allele frequencies (MAF) and different parameters of forensic efficiency—such as power of discrimination (PD), random matching probability (PM), power of exclusion (PE), polymorphism information content (PIC), typical paternity index (TPI), and heterozygosity (He)—were estimated for each locus using GenAlEx software V.6.503 **[12].** Fisher’s exact tests to evaluate the Hardy–Weinberg equilibrium (HWE) by locus and linkage disequilibrium (LD) between pair of loci were estimated with STRAF—A convenient online tool for STR data evaluation in forensic genetics **[13].** Phylogenetic tree was constructed from allele frequency data by using the neighbor-joining method **[14]** *via* web version of POPTREEW **[15]** It is used to compare between different genetic structure of the populations with Bahraini population using the minimum available loci for different populations. The tree was constructed with allele frequency data of fifteen STR loci (D8S1179, D21S11, D7S820, CSF1PO, D19S433, vWA, TPOX, D18S51, D5S818, FGA, D3S1358, TH01, D13S317, D16S539 and D2S1338) for all populations.

Also, Multidimensional scaling (MDS) analysis was done using IBM SPSS Statistics 21.0 **[16]** to investigate the populations structure between Bahraini population and the abovementioned populations based on FST’s genetic distances.

## Results

### Hardy-Weinberg equation (HWE) and linkage disequilibrium (LD)

**Fig 1:**
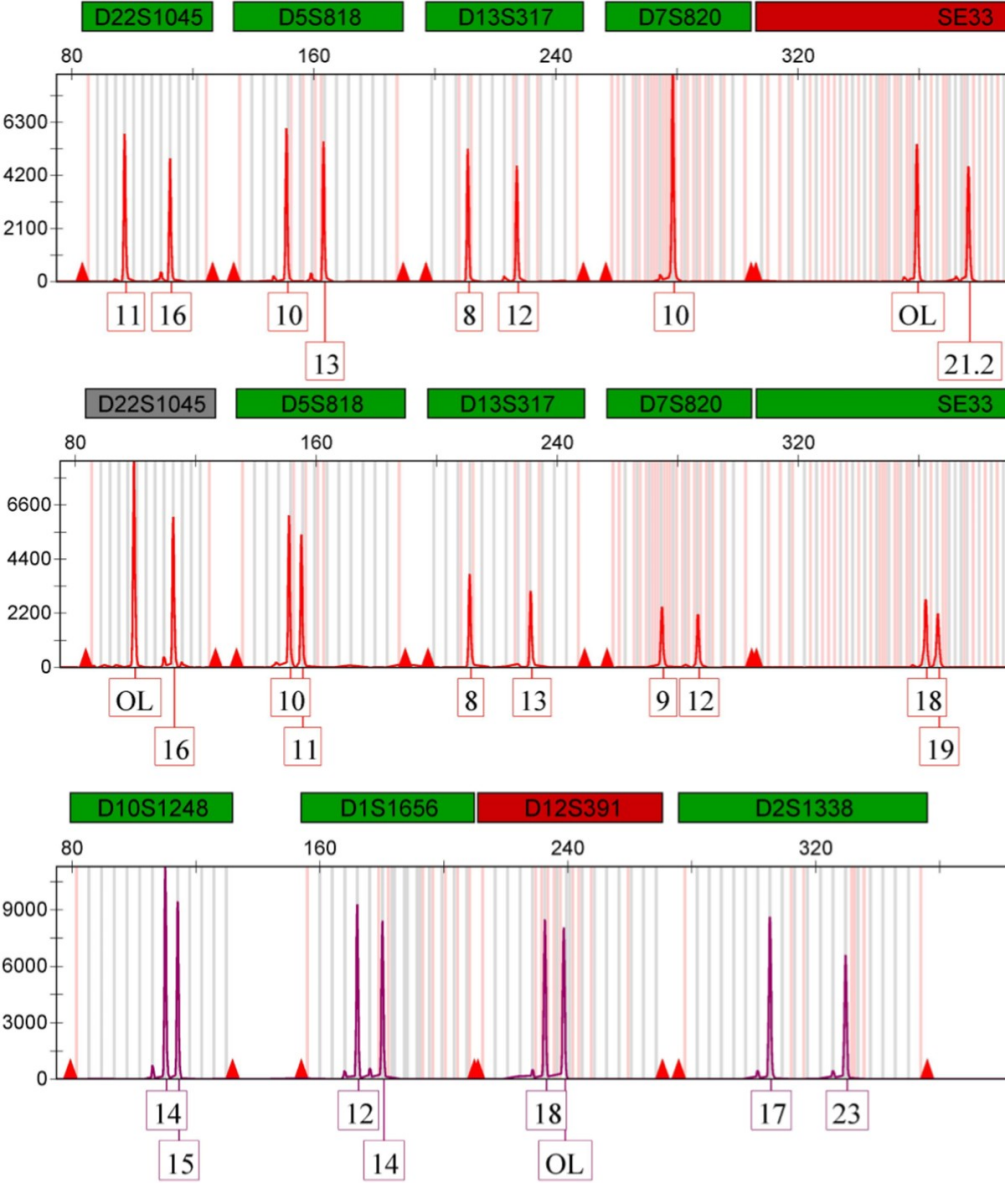
Off-ladder cases observed in D22S0145, SE33 and D12S391.

In the present study no significant deviation from HWE was observed (p> 0.05) except for three markers; D3S1358, D19S433 and D5S818 **(Tables 1–5).** After Bonferroni’s correction was applied (p > 0.000092), all of the samples were in HWE. Full dataset of Bahraini population is shown in **S1 Table**. The study also showed no significant deviation from LD between pairwise STR loci after Bonferroni’s correction (p > 0.000092) in Bahraini population except for the following loci; D3S1358-CSF1PO, CSF1PO-SE33, D19S433-D12S391, FGA-D2S1338, FGA-SE33, FGA-D7S820 and D7S820-SE33 when plotted. The highest pairwise LD was 1.00 when plotting CSF1PO- D19S433, D21S11-FGA and FGA- D1S1656. The marker D22S1045 did not show any probability. This lack of probability correlated with the off-ladder cases observed in D22S1045 and which may be the reason for the null probability value. D22S1015, SE33 and D21S11 loci also revealed evidence of a rare variant and off-ladders **(Fig 1).**

**Table 1:** Allele frequency distribution and statistical parameters of forensic importance for 21 autosomal STRs loci (GlobalFiler kit) in 543 unrelated Bahraini individuals.

Allele	D3S1358	VWA	D16S539	CSF1PO	TPOX	D8S1179	D21S11	D18S51	D2S441	D19S433	TH01	FGA	D22S1045	D5S818	D13S317	D7S820	SE33	D10S1248	D1S1656	D12S391	D2S1338
**6**					0.01197						0.27624			0.00092							
**6.3**																	0.00645				
**7**				0.00737	0.00368						0.24217			0.00184	0.00737	0.02947					
**8**			0.03039	0.01473	0.49632	0.00645					0.12523			0.02026	0.12707	0.18416	0.00737				
**9**			0.13996	0.02302	0.14825	0.00645			0.00368		0.21363			0.06262	0.04880	0.07090	0.00184	0.01013			
**9.1**				0.00092												0.00092					
**9.3**											0.12983										
**10**			0.08379	0.27808	0.07182	0.06077		0.00552	0.14549	0.00368	0.01197		0.00830	0.09853	0.05985	0.28453	0.00092	0.00092	0.00184		
**10.3**											0.00092										
**11**		0.00092	0.37569	0.30847	0.24125	0.09300		0.02210	0.35175	0.02210			0.14945	0.27532	0.25967	0.22284	0.00184	0.01473	0.09576		
**11.2**										0.00092							0.00092				
**11.3**									0.05985												
**12**			0.19521	0.30295	0.02670	0.14457		0.12155	0.08564	0.09024			0.01937	0.35267	0.33702	0.16759	0.00276	0.03131	0.13076		
**12.2**										0.01289							0.00184				
**12.3**									0.00737												
**13**	0.00276	0.00552	0.16114	0.05801		0.24678		0.14825	0.02394	0.21271			0.00554	0.17495	0.12431	0.02762	0.01657	0.21455	0.08471		
**13.2**								0.00184		0.02670							0.00368				
**14**	0.07182	0.08103	0.01381	0.00552		0.22652		0.20994	0.28361	0.24033			0.06919	0.01105	0.03315	0.01105	0.02578	0.30847	0.11971		0.00184
**14.2**										0.06169							0.00460				
**14.3**																			0.00368		

**Table 2:** Allele frequency distribution and statistical parameters of forensic importance for 21 autosomal STRs loci (GlobalFiler kit) in 543 unrelated Bahraini individuals.

Allele	D3S1358	VWA	D16S539	CSF1PO	TPOX	D8S1179	D21S11	D18S51	D2S441	D19S433	TH01	FGA	D22S1045	D5S818	D13S317	D7S820	SE33	D10S1248	D1S1656	D12S391	D2S1338
**15**	0.23849	0.17127		0.00092		0.15838		0.11326	0.03867	0.14088			0.41697	0.00184	0.00276	0.00092	0.01565	0.23665	0.16943	0.02394	
**15.2**										0.09300							0.00184				
**15.3**																	0.00829		0.03959		
**16**	0.29834	0.25506				0.04236		0.13812		0.05709		0.00092	0.25830				0.05709	0.13352	0.19337	0.02394	0.03223
**16.1**												0.00092									
**16.2**										0.02670							0.00092				
**16.3**																			0.04236		
**17**	0.25599	0.25322				0.01381		0.10129		0.00184		0.00276	0.06919				0.08564	0.04236	0.04788	0.13444	0.18508
**17.2**								0.00092		0.00645											
**17.3**																			0.03959	0.00368	
**18**	0.11602	0.17403				0.00092		0.06169				0.01013	0.00277				0.09024	0.00737	0.00460	0.19337	0.10221
**18.2**										0.00276		0.00092									
**18.3**																			0.01473	0.00737	
**19**	0.01381	0.04972						0.04696				0.06906					0.08748		0.00092	0.10958	0.15009
**19.1**																				0.00184	
**19.2**												0.01013					0.00184				
**19.3**																			0.01013	0.00276	
**20**	0.00276	0.00921						0.01473				0.08656	0.00092				0.05157			0.11050	0.15193
**20.2**												0.00092					0.00460				

**Table 3:** Allele frequency distribution and statistical parameters of forensic importance for 21 autosomal STRs loci (GlobalFiler kit) in 543 unrelated Bahraini individuals.

Allele	D3S1358	VWA	D16S539	CSF1PO	TPOX	D8S1179	D21S11	D18S51	D2S441	D19S433	TH01	FGA	D22S1045	D5S818	D13S317	D7S820	SE33	D10S1248	D1S1656	D12S391	D2S1338
**20.3**																			0.00092	0.00092	
**21**								0.00552				0.15285					0.02855			0.13352	0.06077
**21.1**																	0.00276				
**21.2**												0.00829					0.01565				
**22**								0.00737				0.15101					0.01105			0.10497	0.04696
**22.2**												0.00921					0.02210				
**23**								0.00092				0.18416					0.00184			0.08379	0.11971
**23.2**												0.00276					0.01934				
**23.3**												0.00092									
**24**												0.15654					0.00092			0.03959	0.08471
**24.2**												0.00368					0.02670				
**25**												0.09024								0.02118	0.05341
**25.2**																	0.04052				
**26**							0.00184					0.04144								0.00460	0.01013
**26.2**												0.00092					0.04696				
**27**							0.02855					0.01013									0.00092
**27.2**																	0.05157				
**28**							0.16298					0.00276									
**28.2**																	0.05617				
**29**							0.24217					0.00184									

**Table 4:** Allele frequency distribution and statistical parameters of forensic importance for 21 autosomal STRs loci (GlobalFiler kit) in 543 unrelated Bahraini individuals.

Allele	D3S1358	VWA	D16S539	CSF1PO	TPOX	D8S1179	D21S11	D18S51	D2S441	D19S433	TH01	FGA	D22S1045	D5S818	D13S317	D7S820	SE33	D10S1248	D1S1656	D12S391	D2S1338
**29.2**							0.00092										0.04972				
**30**							0.18140					0.00092									
**30.2**							0.02762										0.05709				
**31**							0.05157														
**31.2**							0.10221										0.04144				
**32**							0.00737										0.00092				
**32.2**							0.14088										0.02394				
**33**																	0.00092				
**33.1**							0.00092														
**33.2**							0.03315										0.00645				
**34**							0.00368										0.00092				
**34.2**							0.00737										0.00829				
**35**							0.00184										0.00184				
**35.2**							0.00092														
**36**							0.00184										0.00368				
**37**							0.00276										0.00092				

**Table 5:** Allele frequency distribution and statistical parameters of forensic importance for 21 autosomal STRs loci (GlobalFiler kit) in 543 unrelated Bahraini individuals.

Allele	D3S1358	VWA	D16S539	CSF1PO	TPOX	D8S1179	D21S11	D18S51	D2S441	D19S433	TH01	FGA	D22S1045	D5S818	D13S317	D7S820	SE33	D10S1248	D1S1656	D12S391	D2S1338
MAF* 0.0046 0.0046			0.0046	0.0046	0.0046	0.0046	0.0046	0.0046	0.0046	0.0046	0.0046	0.0046	0.0046	0.0046	0.0046	0.0046	0.0046	0.0046	0.0046	0.0046	0.0046
Forensic parameters**																					
**PM**	0.9068	0.9334	0.9136	0.8786	0.8441	0.9477	0.9580	0.9682	0.9105	0.9591	0.9206	0.9689	0.8886	0.8967	0.9201	0.9290	0.9937	0.9139	0.9726	0.9738	0.9720
**PD**	0.9068	0.9334	0.9136	0.8786	0.8441	0.9477	0.9580	0.9682	0.9105	0.9591	0.9206	0.9689	0.8886	0.8967	0.9201	0.9290	0.9937	0.9139	0.9726	0.9738	0.9720
**PE**	0.5841	0.5410	0.4996	0.4965	0.3839	0.6358	0.6570	0.7001	0.4660	0.6784	0.5410	0.7703	0.4532	0.5153	0.5508	0.6080	0.8381	0.5977	0.6965	0.6965	0.7258
**TPI**	2.4027	2.1548	1.9532	1.9393	1.5168	2.7704	2.9511	3.3938	1.8100	3.1570	2.1548	4.4508	1.7597	2.0261	2.2073	2.5613	6.3140	2.4908	3.3519	3.3519	3.7192
**PIC**	0.7324	0.7730	0.7353	0.6829	0.6215	0.8051	0.8282	0.8566	0.7271	0.8349	0.7531	0.8596	0.6877	0.7173	0.7500	0.7718	0.9466	0.7489	0.8667	0.8696	0.8661
**Ho**	0.7919	0.7680	0.7440	0.7422	0.6703	0.8195	0.8306	0.8527	0.7238	0.8416	0.7680	0.8877	0.7159	0.7532	0.7735	0.8048	0.9208	0.7993	0.8508	0.8508	0.8656
**HWE**	0.0230	0.4470	0.3390	0.3210	0.3220	0.4790	0.2910	0.1490	0.2680	0.0350	0.3100	0.6620	0.2200	0.0230	0.3060	0.2420	0.3720	0.1380	0.5960	0.4300	0.1350

* MAF: Minimum allele frequency based upon the formula 5/2N (N = total number of individuals studied) from NRC recommendations

** PM: random matching probability; PD: power of discrimination; PE: power of exclusion; TPI: typical paternity index; PIC: polymorphism information content; Ho: observed heterozygosity; HWE: Hardy–Weinberg equilibrium test (p>0.05)

### Allele frequencies and forensic parameters

In the studied population, the number of allele (Na) per locus was ranged from 7 for markers D16S539, TPOX and THO1 to 48 for SE33, the mean number of alleles per locus was 14, and a total number of alleles observed was 288. The most polymorphic locus was SE33 **(Tables 1–5).**

The probability that two randomly chosen person have the same unspecified genotype at a locus is the sum squares of the frequencies of all genotypes at that locus. Some alleles show very high frequencies in the Bahraini population; allele 8 in locus TPOX scored the highest frequencies of 0.496 followed by allele 15 in D22S1045 with frequency of 0.417 and the lowest allele frequency was 0.00092 for 35 different alleles. **(Tables 1–5).**

Generally, the polymorphism degree of a specific locus can be measured by two distinct parameters–the heterozygosity and the Polymorphism Information Content (PIC). We have found out that the observed heterozygosity (Ho) was ranged from 67% for locus TPOX to 92% for locus SE33. **(Tables 1–5).**

PIC values for all STR loci were highly informative (PIC≥0.6) with an average of 78.3%. The means for (Na) and (He) designate the high levels of genetic diversity in the population studied. These high informative values support the heterozygosity values indicating the high degree of genetic polymorphism.

The random matching probability (PM) was ranged from 0.006 for SE33 to 0.156 for TPOX. The Power of exclusion (PE) was ranged from 0.384 for locus TPOX to 0.838 for locus SE33. The SE33 locus showed the greatest (PD) in Bahraini population, whereas TPOX showed the lowest. The higher the discrimination power of a locus, the more efficient it is in discriminating between members of the population **(Tables 1–5).**

The PD values for most of the tested loci was above 0.9; the highest value was observed for SE33 (0.994) whereas the least value was observed at TPOX (0.844). The combined power of discrimination (CPD) and combined matching probability (CMP) for all the 21 STR loci were 99.999999% and 4.5633E-27 respectively.

### Interpopulation diversity

**Fig 2:**
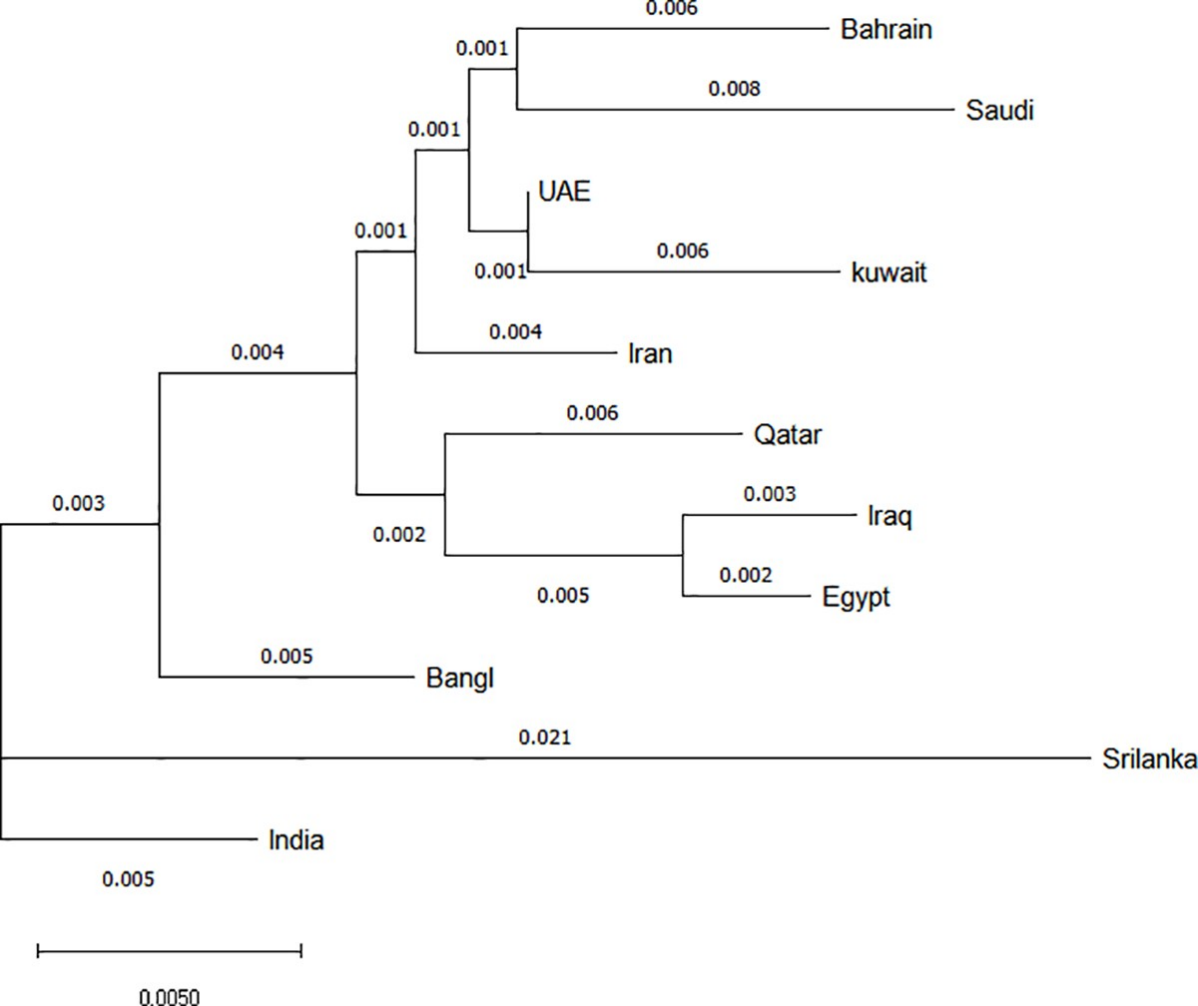
Phylogenetic tree including the FST values, showing the relationship of Bahraini population and other neighboring populations. Qatari population [17], Kuwaiti population [18], Iraqi populations [18], Iranian populations [18], Egyptian population [18], Bengali population [18], Sri Lankan population [18], Indian population [18], Emirati population [19] and Saudi population [18]. The tree was constructed with allele frequency data of fifteen STR loci (D8S1179, D21S11, D7S820, CSF1PO, D19S433, vWA, TPOX, D18S51, D5S818, FGA, D3S1358, TH01, D13S317, D16S539 and D2S1338) for all populations.

**Fig 3:**
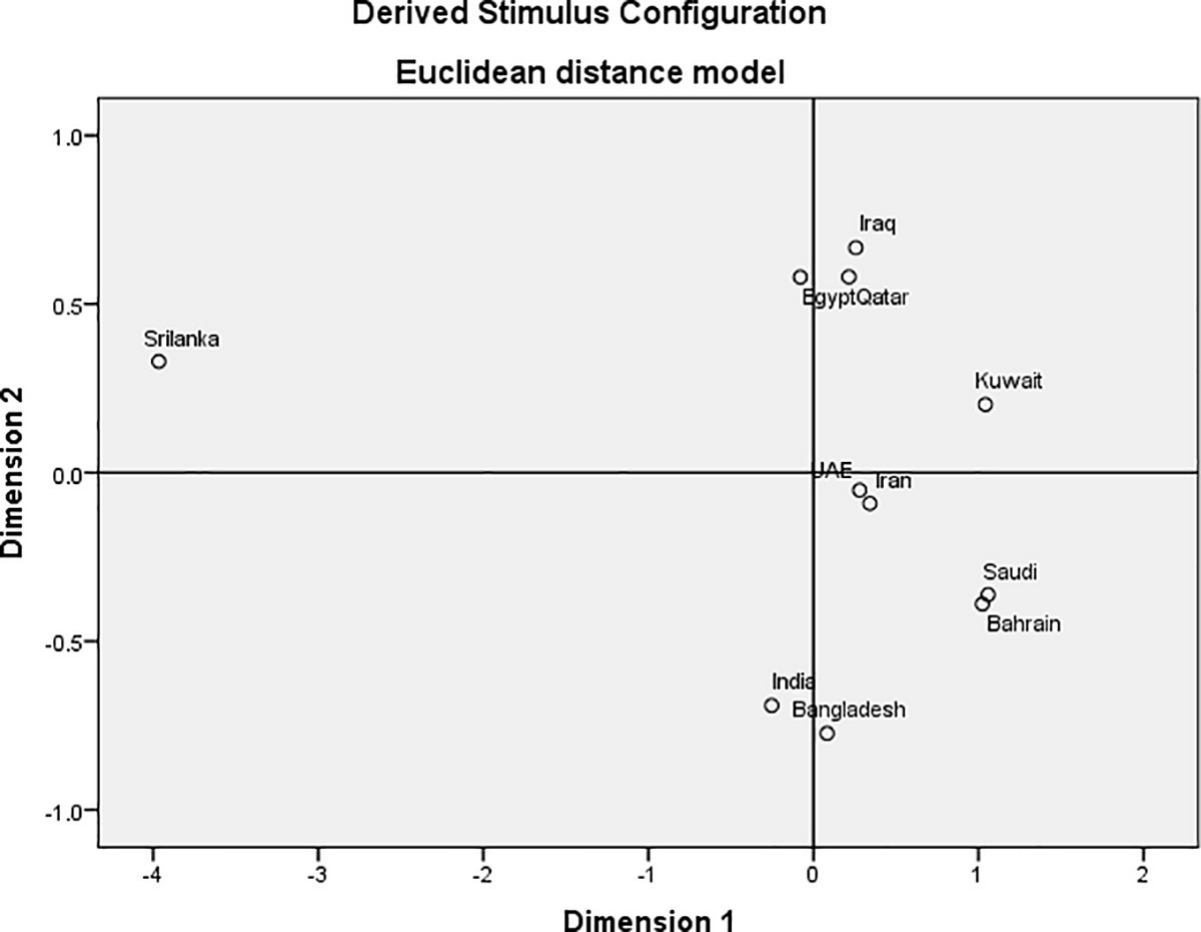
**Multidimensional scaling (MDS) plots of the Bahraini population and other 10 populations; including Qatari population [17], Kuwaiti population [18], Iraqi populations [18], Iranian populations [18], Egyptian population [18], Bengali population [18], Sri Lankan population [18], Indian population [18], Emirati population [19] and Saudi population [18] built using IBM SPSS Statistics v21.0 software based on the Nei’s genetic distances.** For matrix, Stress = .09055 RSQ = .98217.

To measure the diversity between Bahraini population and populations previously reported, phylogenetic tree and MDS were conducted between Qatari population **[17]**, Kuwaiti population **[18]**, Iraqi populations **[18]**, Iranian populations **[18]**, Egyptian population **[18]**, Bengali population **[18]**, Sri Lankan population **[18]**, Indian population **[18]**, Emirati population **[19]** and Saudi population **[18]** based on fixation index FST and Nei’s genetic distances respectively. The comparison with published data showed that the populations in this study had similar pairwise FST values with those populations that are geographically most. As shown in **Fig 2**, Bahraini and Saudi populations share the most genetic relatedness among the other populations, followed by Emirati, Kuwaiti, Iranian, and Qatari in the same cluster. On the other hand, Sri Lankan, Bengali and Indian showed relatedness with each other and in distant genetic structure from Bahraini population. Sample bias corrected FST distances were obtained and were represented multidimensional scaling (MDS) plots (**Fig 3).** As shown, Bahraini and Saudi populations positioned in the right bottom cluster, Bengali and Indian populations clustered together, Emirati and Iranian were also clustered together, followed by Iraqi, Qatari, and Egyptian in the same cluster. Sri Lankan and Kuwaiti populations were in separate clusters found apart.

### Rare variants, off-ladder and null alleles

**Fig 4:**
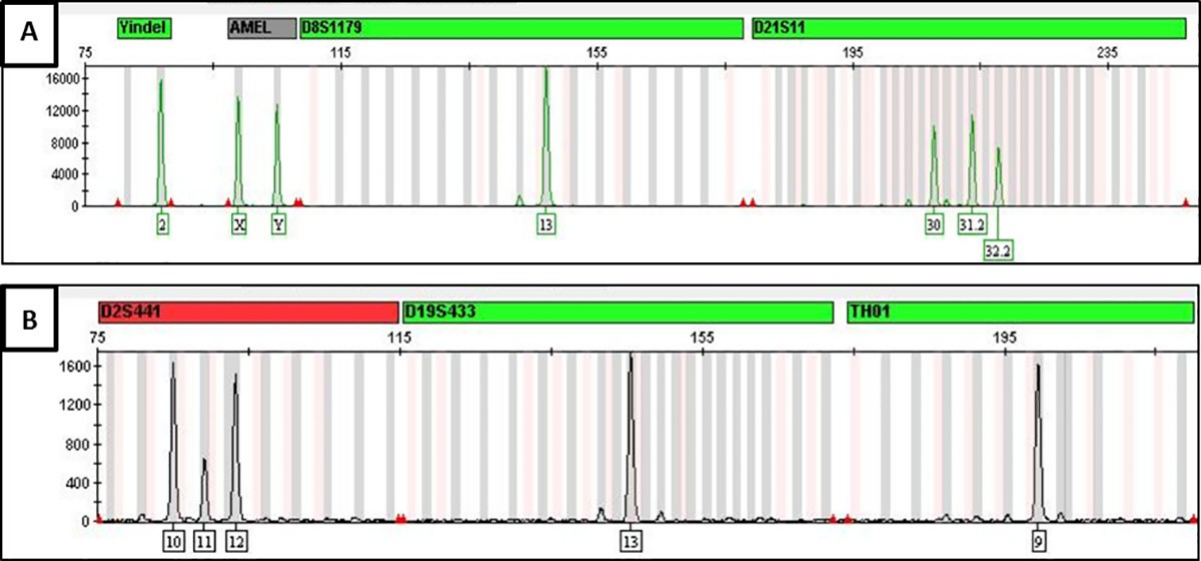
Two electropherograms (A & B) indicating the tri-allelic patterns.

Different samples showed off ladder (OL) in 10 various cases; two allelic ladder variants were detected at the D12S391, Sample#5 indicated OL (18,OL) in 238.69 bp and sample#511 showed OL (OL,21) in 238.64 bp. Two allelic ladder variants were detected at the SE33, Sample#288 indicated OL in (OL,23.2) in 320.61 bp and Sample#538 with OL (OL,21.2) in 359.20 bp. Six allelic ladder variants were detected in D22S1045, sample#309 showed OL (OL,17) in 99.44 bp, sample#331 showed OL (OL,15) in 99.51 bp, sample#487 showed another OL (OL,14) in 99.45 bp, sample#516 indicated an OL (OL,16) in 99.44 bp, sample#524 showed OL (OL,14) in 99.45 bp and sample #549 showed OL (OL,16) in 99.44 bp **(Fig 1).** As for the tri-allelic patterns, sample#180 showed three variants in D21S11 (30,31.2,32.2) with sizes of 207.67 bp, 213.64 bp and 217.78 bp **(Fig 4A)** respectively and it was not observed and reported in STRbase (http://strbase.nist.gov/index.htm) **[20].** Sample# 520 showed 3 variants in D2S441 (10,11,12) with sizes of 85.03 bp, 89.15 bp and 93.30 bp respectively (**Fig 4B)**. Whereas the adjacent locus D19S433 was of homozygous allele (13,13) and it was observed and previously reported in STRbase (http://strbase.nist.gov/index.htm) **[20].**

## Discussion

The observed deviation from HWE (neglecting the Bonferroni’s correction) could be a result of the diversity of the Bahraini population or caused by high polymorphism at the same loci investigated loci. This observation are likely to reflect the high level of inbreeding with consanguinity rates in Bahrain, with intra-familial unions accounting for 20–50% of all marriages compared to other Arab countries **[21]**. The PD in correlation with PM supports the high degree of polymorphism between Bahraini individuals.

We have compared Bahraini population data to the nearest available populations using the accessible loci. It is shown that the Bahraini population shares similar results with the study conducted of Saudi Arabia and UAE populations using the GlobalFiler STR loci **[19, 22]**. As the above-mentioned populations share the most informative and polymorphic locus is SE33 and the least informative locus is TPOX. The least polymorphic was locus D16S539 for UAE population **[19]** whereas THO1 for both Bahraini and Saudi Arabian populations **[22].** Allele 8 in locus TPOX scored the highest frequency for Bahraini, Kuwaiti, Saudi Arabian, Iraqi, Egyptian and Iranian populations **[18, 22]** whereas the highest frequency for Indian and Bangladeshi populations is allele 12 in CSF1PO **[18].** Regarding the phylogenetic tree construct, the data from the ten populations are consistent with other population data from the region **[18, 23, 24]** based upon the FST values obtained. The obtained FST value of Bahrain is 0.006 which is less than the recommended value for casework statistics of FST < 0.01 **[25]**.

As expected, the diversity between the data obtained in this study compared to the neighboring data populations varies, as the populations become more geographically separated.

Once more studies of Arab populations in the region become accessible, it may be more probable to develop a greater understanding of the genetic associations between the different populations for the Arabian Peninsula.

## Conclusions

In conclusion, we have reported the allele frequencies and forensic statistical parameters of the GlobalFiler STR loci in Bahraini population to be indicated in literature for the first time. The polymorphism of the 21 autosomal markers observed in this study such as SE33 marker indicates its usefulness for paternity testing, forensics and familial DNA searching in the population of Bahrain.

Overall, these parameters indicated the general utility of this STR loci panel for forensic personal identification and paternity testing in the Bahraini population, thereby further confirming of its efficacy for forensic practice also in Bahraini sub-populations and other populations' genetics and diversity studies.

## Supporting information

S1 Table Bahrain population dataset. (XLSX)
